# Current status of multilayer neutron interferometry with gaseous samples at J-PARC

**DOI:** 10.1107/S1600576726002931

**Published:** 2026-05-14

**Authors:** Taro Nambu, Clayton J. Auton, Takuhiro Fujiie, Masahiro Hino, Takuya Hosobata, Go Ichikawa, Masaaki Kitaguchi, Anna L. McElhannon, Kenji Mishima, Yoshichika Seki, Hirohiko M. Shimizu, William M. Snow, Yutaka Yamagata

**Affiliations:** a Department of Physics Nagoya University,Nagoya 464-8602 Aichi Japan; b RIKEN Center for Advanced Photonics, Wako 351-0198, Saitama, Japan; c Indiana University, Bloomington, Indiana 47401, USA; d Department of Physics, Rikkyo University, Toshima 171-8501, Tokyo, Japan; e Institute for Integrated Radiation and Nuclear Science, Kyoto University, Kumatori 590-0494, Osaka, Japan; f High Energy Accelerator Research Organization, Tokai Ibaraki 319-1106 Japan; g J-PARC Center, 2-4 Tokai, Ibaraki 319-1165, Japan; h Kobayashi-Maskawa Institute Nagoya University,Nagoya 464-8602 Aichi Japan; i Research Center for Nuclear Physics, University of Osaka, Ibaraki, Osaka 567-0047, Japan; j Institute of Multidisciplinary Research for Advanced Materials, Tohoku University, Aoba, Sendai 980-8577, Japan; Lund University, Sweden

**Keywords:** few-body systems, neutron interferometers, scattering lengths

## Abstract

Several few-body nuclear models, such as the Argonne *v*_18_ potential including three-nucleon forces and chiral effective field theories, predict the coherent scattering length (*b*_c_) for light nuclei. Reliable measurements of *b*_c_ therefore provide important tests of these models. We have developed a measurement system for determining *b*_c_ of gaseous samples using a multilayer-type neutron interferometer at J-PARC, and successfully observed the phase shifts of ^3^He and ^4^He gases. From the observed phase shifts, we obtained for ^3^He *b*_c_ = 3.99 ± 0.23 (stat.) ± 0.66 (sys.) fm and for ^4^He *b*_c_ = 2.95 ± 0.11 (stat.) ± 0.67 (sys.) fm (stat. and sys. denote statistical uncertainty and systematic uncertainty, respectively). To achieve higher precision, we are currently upgrading the experimental setup.

## Abstract 2

First measurements of the neutron scattering lengths of ^3^He and ^4^He using a multilayer-type neutron interferometer at J-PARC are reported, demonstrating the initial feasibility of the method with prospects for improved precision.

## 1. Introduction

The coherent scattering length (*b*_c_) describes neutron s-wave scattering and is a key parameter for understanding few-body nuclear systems. Few-body nuclei such as ^3^He and ^4^He are described by several phenomenological models, including the Argonne *v*_18_ (AV18) potential with three-nucleon interactions (3*N*), the *R* matrix approach (Hofmann & Hale, 2003 ▸) and chiral effective field theories (Bagnarol *et al.*, 2023 ▸), which predict *b*_c_.

Therefore, measurements of the *b*_c_ of ^3^He and ^4^He (denoted hereafter as 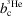 and 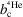) can serve as benchmarks for studies of few-body nuclear systems.

Experimentally, *b*_c_ values have been measured using several methods, including scattering cross-section measurements (Haddock *et al.*, 2019 ▸), reflectometry (Kitchens *et al.*, 1974 ▸), diffraction (Shull & Shaw, 1973 ▸) and, most prominently, neutron interferometry (Ioffe *et al.*, 1998 ▸). For instance, the *b*_c_ values of H and D, obtained using neutron interferometers (NIs) with relative precisions of 3.0 × 10^−4^ (Koester & Nistler, 1975 ▸) and 4.5 × 10^−4^ (Schoen *et al.*, 2003 ▸), respectively, are consistent with the predictions of AV18 and AV18 with 3*N*. However, relative discrepancies of approximately 2 × 10^−2^ for 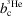 and 7 × 10^−3^ for 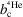 persist among the latest measurements using Si crystal NIs.

We aim to resolve these discrepancies with a relative precision of 10^−3^ using pulsed neutrons and a multilayer-type NI. While *b*_c_ for solid samples has already been measured using a multilayer-type NI, this paper reports its first application to gaseous samples with a newly fabricated gas cell. We present the results as a proof of principle for this method and discuss developments towards higher-precision experiments.

## 2. Experiment

A neutron interferometer splits a neutron wave into two paths and recombines them to produce interference. The potential difference between the two paths appears as a phase shift in the interference fringes. Since the first implementation in 1974 (Rauch *et al.*, 1974 ▸), NIs have found applications across diverse domains of physics (Colella *et al.*, 1975 ▸; Rauch *et al.*, 1975 ▸; Hasegawa *et al.*, 2003 ▸).

The phase shift Δϕ is described by 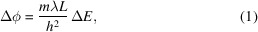
where *m* is the neutron mass, λ is the neutron wavelength, *L* is the interaction length with the potential, Δ*E* is the potential difference between the two paths and *h* is Planck’s constant. The sensitivity of an NI is proportional to λ and *L*. Consequently, extending the path length of an NI and using a long wavelength enhances its sensitivity. A conventional NI made from an Si single crystal relies on Bragg scattering, which prevents its operation at λ longer than the Si lattice spacing. The interaction length *L* and the size of the NI are constrained by the dimensions over which high crystallinity can be maintained.

### 2.1. Multilayer neutron interferometer

**Figure 1:**
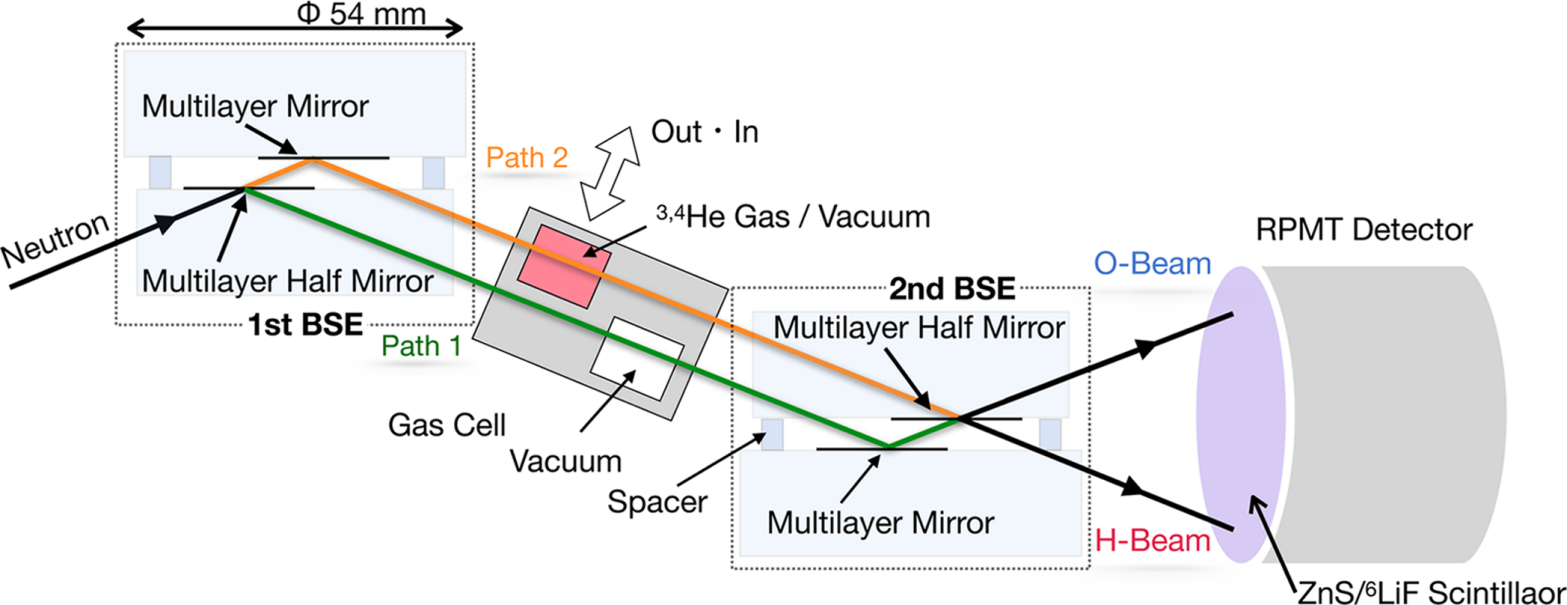
Experimental setup of the Jamin-type NI employing beam-splitting etalons (BSEs).

We developed an NI using multilayer neutron mirrors. A multilayer neutron mirror consists of periodic layers of two materials (Ni/Ti) with different Fermi pseudo-potentials for neutrons. We can utilize cold neutrons which satisfy the reflection condition. Because the layer spacing is larger than the lattice spacing of an Si single crystal, neutrons with longer λ can be utilized than in a conventional NI. For the application of multilayer mirrors to a Jamin-type NI (shown in Fig. 1 ▸), we used a pair of beam-splitting etalons (BSEs) (Kitaguchi *et al.*, 2003 ▸; Seki *et al.*, 2010 ▸). Each BSE is composed of two multilayer mirrors on SiO_2_ substrates, separated by an air gap of 211 µm.

The NI with BSEs not only allows the extension of *L* but also enables the insertion of large samples that would be difficult to accommodate in a conventional NI, expanding the flexibility of the experimental setup. In the present measurement, a gas cell with a total width of 40 mm was inserted between the two paths, providing *L* = 8 mm. The value of *L* is potentially scalable up to 1000 mm.

### 2.2. Experimental setup

We conducted experiments on the low-divergence branch of the J-PARC beamline MLF BL05 (NOP) (Mishima *et al.*, 2009 ▸). The neutron wavelength used by a multilayer-type NI is determined by the time-of-flight (TOF) method, reducing time-varying disturbances in the analysis process. The multilayer-type NI and the sample insertion assembly were placed on a vibration isolation table inside a thermostatic chamber. To ensure the stability and alignment of the mirror substrates, the rotation of the entire NI and the relative angles between the two BSEs were precisely controlled and maintained using high-precision stepping motors. We observed an interferogram using pulsed neutrons (Fujiie *et al.*, 2024 ▸). The bandwidth of λ is from 0.9 to 1.1 nm. The phase determination accuracy was comparable to that of a conventional NI. In our previous work, we had already measured *b*_c_ for several nuclei with solid samples and confirmed that these values were consistent with previous studies, except for V (Fujiie *et al.*, 2024 ▸).

### 2.3. Gas cell

**Figure 2:**
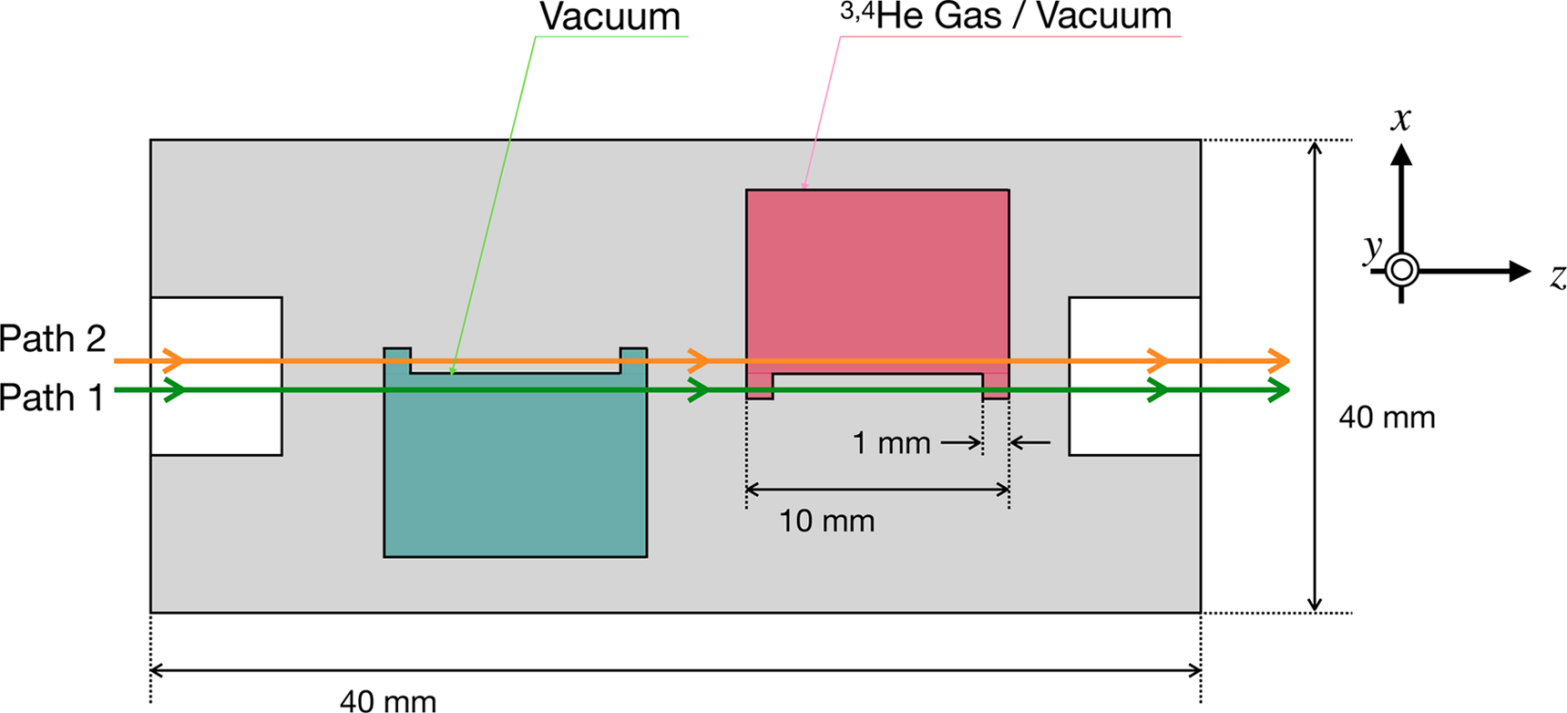
Cross-section view of the gas cell. The red region contains the gaseous sample, while the green region is evacuated.

For measuring the *b*_c_ of gaseous samples, we designed a gas cell (shown in Fig. 2 ▸) which was machined from pure aluminium alloy (A1050) at RIKEN. In *b*_c_ measurements using an NI, one path passes through the sample while the other passes through a vacuum, thereby producing the phase shift. For a conventional NI, the centimetre-scale path separation allows the use of a two-chamber gas cell separated by a partition. However, in a multilayer-type NI, the beam separation is only 380 µm, so neutrons pass through the corner radius formed during fabrication, making it difficult to determine *L* through the gas region accurately.

To address this problem, recess machining was performed to create surfaces perpendicular to the neutron path. By creating a 1 mm extension in the *x*-axis direction, neutrons are directed perpendicularly onto the gaseous sample. Neutrons pass through the red region, 2 mm in Path 1 and 10 mm in Path 2; therefore the effective *L* contributing to the phase shift is 8 mm.

### 2.4. Experiment procedure and analysis

Measurements were repeated with and without the sample to minimize disturbances, as illustrated in Fig. 1 ▸, with mea­sure­ments taken every 10 min. We used a position-sensitive detector with time resolution, consisting of a resistance-division photomultiplier tube (RPMT) and a ZnS/^6^LiF scintillator. The detector was 0.3 m away from the interferometer setup (shown in Fig. 1 ▸). The details are described by Hirota *et al.* (2005 ▸).

The sample gases were filled at 50256 ± 6 Pa at 299.497 ± 0.06 K for ^3^He and at 100017 ± 10 Pa at 299.196 ± 0.06 K for ^4^He, measured by a piezoresistive transducer and a platinum resistance thermometer. The gas cell was evacuated by a turbo molecular pump to 3.00 × 10^−3^ Pa before filling, and the residual vacuum pressures before filling the gases were 1.00 × 10^−2^ Pa for ^3^He and 1.17 × 10^−2^ Pa for ^4^He. Table 1 ▸ sum­marizes the assessment of sample contamination. The levels were below the experimental uncertainty and therefore negligible for these measurements.

**Figure 3:**
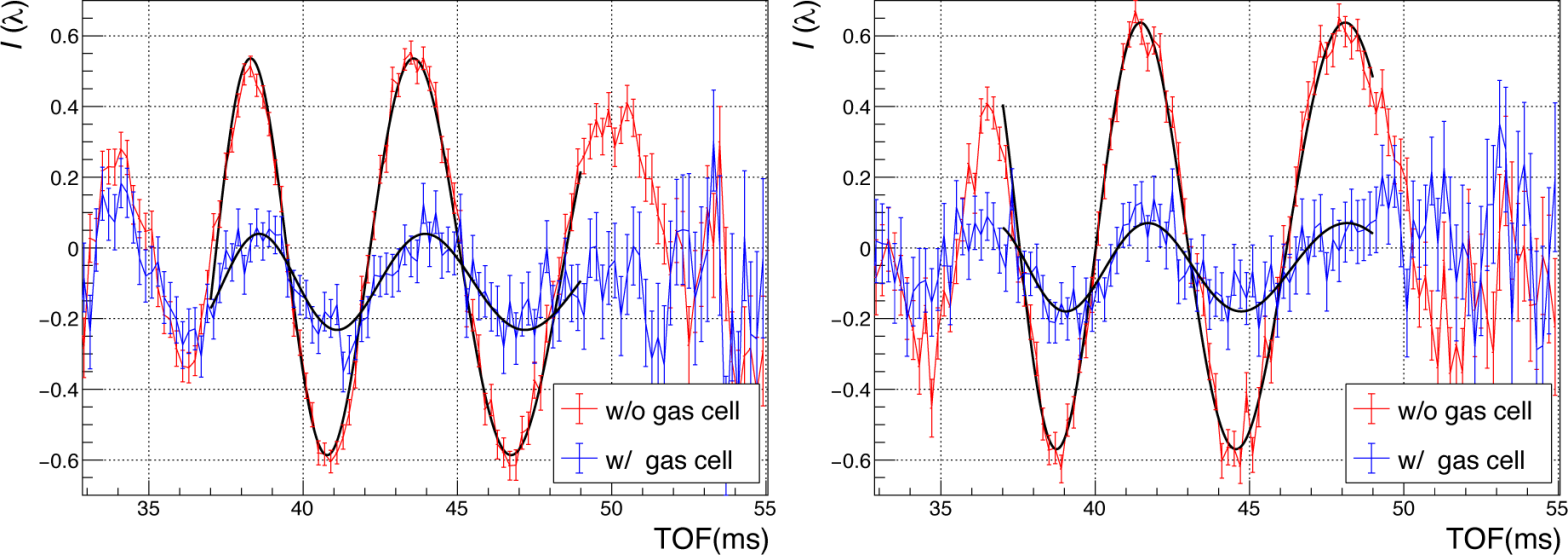
Interference fringes for (left) ^3^He and (right) ^4^He. Red and blue lines correspond to data with the cell out and in, respectively. Black lines indicate fits using equation (3).

**Figure 4:**
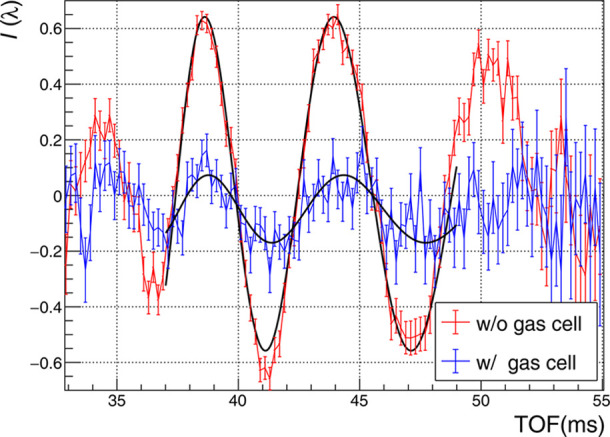
Interference fringes for the double-path vacuum measurement. Red and blue lines correspond to data with the cell out and in, respectively. Black lines indicate fits using equation (3).

Figs. 3 ▸ and 4 ▸ show the interference fringes obtained over 10 min under conditions with the gas cell filled with ^3^He, ^4^He and evacuated, respectively, and with the gas cell inserted and removed from the beam path. In Figs. 3 ▸ and 4 ▸, the vertical axis *I*(λ) is defined by
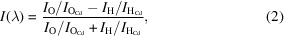
where *I*_H_ and *I*_O_ are the intensities of the O and H beams, respectively, and 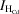 and 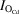 are those with a Cd block inserted. We fitted the obtained interference fringes using the fitting function
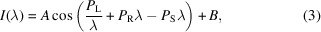
where *A* denotes the contrast of the interferogram. The coefficients *P*_L_ and *P*_R_ represent the inverse proportional and proportional terms, respectively, and they are derived by geometric optics (Fujiie *et al.*, 2024 ▸). Specifically, *P*_R_ accounts for the phase shift contributed by the SiO_2_ substrate. The third term, *P*_S_ = *Nb*_c_*t*, is the interaction with the sample, where *N* is the number density of atoms and *t* is the sample thickness. The TOF region used for the fit was set from 37 to 49 ms, which corresponds to the reflecting momentum transfer range of the multilayer mirror, 0.232 < *Q* < 0.292 nm^−1^. Through relative measurements, *P*_S_ was isolated as the difference between the wavelength-proportional terms with and without the sample. Scattering lengths were calculated from the phase shifts of each sample (shown in Fig. 3 ▸) after subtracting the value obtained in the vacuum measurement (shown in Fig. 4 ▸).

The extracted phase shifts were 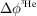
 = 0.38 ± 0.022 (stat.) rad, 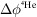 = 0.57 ± 0.022 (stat.) rad and Δϕ^Vac^ = 0.19 ± 0.015 (stat.) rad (where stat. means statistical uncertainty). Consequently, the results were 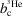
 = 3.99 ± 0.23 (stat.) fm and 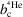 = 2.95 ± 0.11 (stat.) fm, with measurement times of 6 and 8 h, respectively. The relatively large statistical uncertainties were caused by a decrease in fringe contrast. The decrease in contrast caused by the insertion of the cell is attributed to small-angle scattering of neutrons by the 18 mm-thick (in total) aluminium wall (shown in Fig. 2 ▸).

### 2.5. Uncertainties

The systematic uncertainties are summarized in Table 2 ▸. The dominant systematic uncertainty is the phase distribution in the beam cross section, which is described below. In the condition with no cell, the phase of the interferogram in the TOF varied in the vertical position on the detector, indicating that the neutron phase was distributed along the *y* axis. We suspect that this effect was caused by a misalignment of the BSEs. We divided the data into three regions along the *y* axis and analysed each region to derive phase shifts, but the results were inconsistent. The systematic uncertainties arise from the weighted average taken for the three data points, expanding the error bar such that χ^2^/ndf = 1, where χ^2^ is the chi-squared parameter and ndf the number of degrees of freedom.

## 3. Results

**Figure 5:**
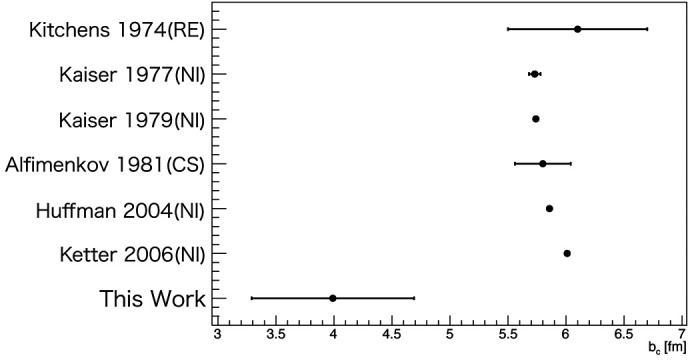
Comparison of measured 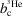 with previous results.

Figs. 5 ▸ and 6 ▸ compare our results with previous work (Kitchens *et al.*, 1974 ▸; Kaiser *et al.*, 1977 ▸; Kaiser *et al.*, 1979 ▸; Alfimenkov *et al.*, 1981 ▸; Huffman *et al.*, 2004 ▸; Ketter *et al.*, 2006 ▸; McReynolds, 1951 ▸; Genin *et al.*, 1963 ▸; Rorer *et al.*, 1969 ▸; Haddock *et al.*, 2019 ▸; Haun *et al.*, 2020 ▸). The methods used were cross section (CS), reflectivity (RE) and neutron interferometer (NI). Our results were 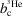
 = 3.99 ± 0.23 (stat.) ± 0.66 (sys.) fm and 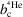
 = 2.95 ± 0.11 (stat.) ± 0.67 (sys.) fm (where sys. means sys­tematic uncertainty). In this first gaseous sample measurement, a significant phase shift was successfully observed, and several issues in the experimental setup for future measurements were identified.

## 4. Discussion

The experimental results revealed a vertical phase distribution and temporal instability, leading to reduced visibility and an underestimation of the phase shift. We recently identified that this distribution stems primarily from the roll angle mis­alignment of the BSEs. Because these systematic effects have not yet been fully quantified, the actual uncertainty in these preliminary results is likely to be larger than that reported above. Furthermore, small-angle scattering from the aluminium degraded the contrast. To address these issues, we are upgrading the setup with high-rigidity stages and low-thermal-expansion materials.

## 5. Future prospects

To overcome the current limitations, we are preparing a new cell using SiO_2_ crystals to eliminate contrast degradation from small-angle scattering. We will also correct the BSE roll angle misalignment by making adjustments, and improve the stability by introducing more rigid components. Furthermore, introducing supermirrors optimized for the J-PARC MLF BL05 spectrum is expected to increase the neutron flux by a factor of 20. With these upgrades, 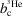 and 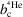 can be measured with a relative precision of 10^−3^ in 6 and 20 h, respectively.

## 6. Summary

To study the few-body system in nuclei, we performed measurements of 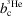 and 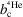 using a multilayer-type NI at J-PARC. The precision of *b*_c_ was limited by statistical uncertainties due to the small-angle scattering from aluminium and setup-related systematic uncertainties. By integrating SiO_2_ cells, a high-rigidity stage and supermirror upgrades, we aim to achieve higher-accuracy measurements.

## Figures and Tables

**Figure 6:**
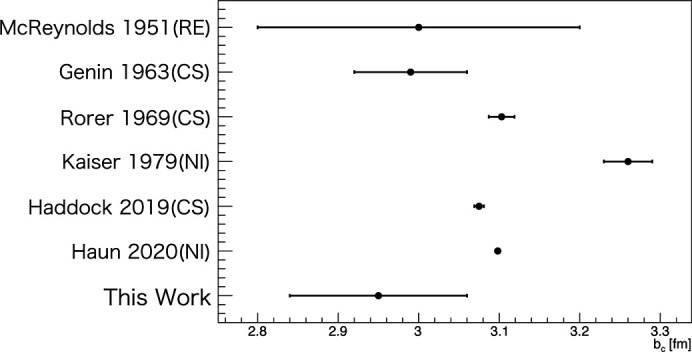
Comparison of measured 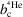 with previous results.

**Table 1:** Impurity list for samples

Sample	Impurity level
^3^He	<10^−3^
^4^He	<10^−6^

**Table 2:** A summary of systematic uncertainties for *b*_c_

	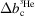 (fm)	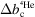 (fm)
Temperature	±0.00079	±0.00059
Pressure	±0.00063	±0.00023
Phase distribution	±0.66	±0.67

## Data Availability

The data are available from the authors upon reasonable request.

## References

[bb1] Alfimenkov, V. P., Borzakov, S. B., Van Tkhuan, V., Govorov, A. M., Lason, L., Pikel’ner, L. B. & Sharapov, E. A. (1981). *Sov. J. Nucl. Phys.* **33**, 467–471.

[bb2] Bagnarol, M., Schäfer, M., Bazak, B. & Barnea, N. (2023). *Phys. Lett. B* **844**, 138078.

[bb3] Colella, R., Overhauser, A. W. & Werner, S. A. (1975). *Phys. Rev. Lett.* **34**, 1472–1474.

[bb4] Fujiie, T., Hino, M., Hosobata, T., Ichikawa, G., Kitaguchi, M., Mishima, K., Seki, Y., Shimizu, H. M. & Yamagata, Y. (2024). *Phys. Rev. Lett.* **132**, 023402. 10.1103/PhysRevLett.132.023402 38277600

[bb5] Genin, R., Beil, H., Signarbieux, C., Carlos, P., Joly, R. & Ribrag, M. (1963). *J. Phys. Radium* **24**, 21–26.

[bb6] Haddock, C. C., Hiromoto, M., Hirota, K., Ino, T., Kitaguchi, M., Mishima, K., Oi, N., Shima, T., Shimizu, H. M., Snow, W. M. & Yoshioka, T. (2019). *Phys. Rev. C* **100**, 064002.

[bb7] Hasegawa, Y., Loidl, R., Badurek, G., Baron, M. & Rauch, H. (2003). *J. Phys. Soc. Jpn* **72**, 42–45.

[bb8] Haun, R., Wietfeldt, F. E., Arif, M., Huber, M. G., Black, T. C., Heacock, B., Pushin, D. A. & Shahi, C. B. (2020). *Phys. Rev. Lett.* **124**, 012501. 10.1103/PhysRevLett.124.012501 PMC8609613 31976711

[bb9] Hirota, K., Shinohara, T., Ikeda, K., Mishima, K., Adachi, T., Morishima, T., Satoh, S., Oku, T., Yamada, S., Sasao, H., Suzuki, J. & Shimizu, H. M. (2005). *Phys. Chem. Chem. Phys.* **7**, 1836. 10.1039/b417838f 19787946

[bb10] Hofmann, H. M. & Hale, G. M. (2003). *Phys. Rev. C.* **68**, 021002.

[bb11] Huffman, P. R., Jacobson, D. L., Schoen, K., Arif, M., Black, T. C., Snow, W. M. & Werner, S. A. (2004). *Phys. Rev. C.* **70**, 014004. 10.1103/PhysRevLett.90.192502 12785940

[bb12] Ioffe, A., Jacobson, D. L., Arif, M., Vrana, M., Werner, S. A., Fischer, P., Greene, G. L. & Mezei, F. (1998). *Phys. Rev. A* **58**, 1475–1479.

[bb13] Kaiser, H., Rauch, H., Badurek, G., Bauspiess, W. & Bonse, U. (1979). *Z. Phys. A* **291**, 231–238.

[bb14] Kaiser, H., Rauch, H., Bauspiess, W. & Bonse, U. (1977). *Phys. Lett. B* **71**, 321–323.

[bb15] Ketter, W., Heil, W., Badurek, G., Baron, M., Jericha, E., Loidl, R. & Rauch, H. (2006). *Eur. Phys. J. A* **27**, 243–256.

[bb16] Kitaguchi, M., Funahashi, H., Nakura, T., Hino, M. & Shimizu, H. M. (2003). *Phys. Rev. A* **67**, 033609.

[bb17] Kitchens, T. A., Oversluizen, T., Passell, L. & Schermer, R. I. (1974). *Phys. Rev. Lett.* **32**, 791–794.

[bb18] Koester, L. & Nistler, W. (1975). *Z. Phys. Teil A* **272**, 189–196.

[bb19] McReynolds, A. W. (1951). *Phys. Rev.* **84**, 969–972.

[bb20] Mishima, K., Ino, T., Sakai, K., Shinohara, T., Hirota, K., Ikeda, K., Sato, H., Otake, Y., Ohmori, H., Muto, S., Higashi, N., Morishima, T., Kitaguch, M., Hino, M., Funahashi, H., Shima, T., Suzuki, J., Niita, T., Taketani, K., Seki, Y. & Shimizu, H. M. (2009). *Nucl. Instrum. Methods Phys. Res. A* **600**, 342–345.

[bb21] Rauch, H., Treimer, W. & Bonse, U. (1974). *Phys. Lett. A* **47**, 369–371.

[bb22] Rauch, H., Zeilinger, A., Badurek, G., Wilfing, A., Bauspiess, W. & Bonse, U. (1975). *Phys. Lett. A* **54**, 425–427.

[bb23] Rorer, D., Ecker, B. & Akyüz, R. (1969). *Nucl. Phys. A* **133**, 410–416.

[bb24] Schoen, K., Jacobson, D. L., Arif, M., Huffman, P. R., Black, T. C., Snow, W. M., Lamoreaux, S. K., Kaiser, H. & Werner, S. A. (2003). *Phys. Rev. C.* **67**, 044005. 10.1103/PhysRevLett.90.192502 12785940

[bb25] Seki, Y., Funahashi, H., Kitaguchi, M., Hino, M., Otake, Y., Taketani, K. & Shimizu, H. M. (2010). *J. Phys. Soc. Jpn* **79**, 124201.

[bb26] Shull, C. G. & Shaw, W. M. (1973). *Z. Naturforsch. Teil A* **28**, 657–661.

